# Evaluation of a semi-automatic isoelectric focusing method for apolipoprotein E phenotyping

**DOI:** 10.1016/j.plabm.2019.e00150

**Published:** 2019-12-17

**Authors:** Randa Bittar, Alain Carrié, Georges Nouadje, Corinne Cherfils, Valérie Fesel-Fouquier, Laurence Barbot-Trystram, Philippe Giral, Dominique Bonnefont-Rousselot

**Affiliations:** a Service de Biochimie Métabolique, AP-HP.Sorbonne Université, F-75013, Paris, France; b Sorbonne Université, UMR_S1166 ICAN, F-75013, Paris, France; c Service de Biochimie Endocrinienne et Oncologique, AP-HP.Sorbonne Université, F-75013, Paris, France; d Sebia, F-91008, Evry, France; e Service de Coprologie Fonctionnelle, AP-HP.Sorbonne Université, F-75013, Paris, France; f Service d’Endocrinologie-Métabolisme, AP-HP.Sorbonne Université, F-75013, Paris, France; g Université de Paris, UTCBS, CNRS, INSERM, F-75006, Paris, France

**Keywords:** Apolipoprotein E, Isoforms, Phenotype, Isoelectric focusing, apo, apolipoprotein, HDL, high density lipoprotein, IDL, intermediate density lipoprotein, IEF, isoelectric focusing, LDL, low density lipoprotein, VLDL, very low density lipoprotein

## Abstract

A qualitative, semi-automatized method for apolipoprotein E (apoE) phenotyping by isoelectric focusing method has been evaluated on 40 serum samples from patients previously genotyped for apoE, especially as regards concordance with genotyping, but also repeatability and reproducibility of the method, and sample storage. Total concordance with genotyping and good precision criteria, together with its practicability and requirement of a little sample volume, lead to conclude to the usefulness of this method to help clinicians in the diagnosis of dyslipidemic and neurodegenerative diseases.

## Highlights

• Semi-automatic isoelectrofocusing method for apoE phenotyping.
• Total concordance with genotyping.
• Good repeatability and reproducibility.

## 1 Introduction

Apolipoprotein E (apoE) is a 34-kDa glycoprotein of 299 amino acids, encoded by the human *APOE* gene located on chromosome 19. Three alleles of this gene occur at different frequencies in humans: ε2 (5–10%), ε3 (65–70%) and ε4 (15–20%), leading to three homozygous (apoE2/E2, apoE3/E3, apoE4/E4) and three heterozygous (apoE3/E2, apoE4/E2, apoE4/E3) phenotypes [1]. The three isoforms produced, i.e., apoE2 (cys112, cys158), apoE3 (cys112, arg158) and apoE4 (arg112, arg158), differ by the structure (conformation, isoelectric point, molecular weight) and by their functional properties [2]. Plasma apoE mainly arises from liver hepatocytes (75%), but the brain (astrocytes, oligodendrocytes, microglia, astrocytes) also synthesizes apoE found in the cerebrospinal fluid. ApoE is a major component of lipoproteins which participates in the transport and clearance of lipids. ApoE4 status is a risk factor for Alzheimer’s and other neurodegenerative diseases, whereas apoE2 and also apoE4 increase the risk for cardiovascular disease [1].

From a practical standpoint, determination of apoE status using isoelectric focusing (IEF) can be easier than genotyping (the latter requiring a genetic consent) and thus constitutes a valuable alternative to genotyping. Initial IEF methods required isolation of lipoproteins (i.e, very low density lipoproteins, VLDLs, and intermediate density lipoproteins, IDLs) by ultracentrifugation and their delipidation before performing isoelectric focusing in a pH 4–6 ampholine gradient [3]. A two-dimensional electrophoresis [4] could further allow confirming E2/E2 phenotype or detection of a mutated form of apoE. Despite that the apoE phenotype revealed by isoelectric focusing was established with more than 20 years ago, a kit commercially available for clinical laboratories does not exist. More recently, a semi-automatic method has been developed that can be performed directly on serum and that was stated as concordant with mass spectrometry [5]. The present study aimed at evaluating this simple qualitative, semi-automatic apoE IEF method on selected samples from patients previously genotyped for apoE, especially regarding concordance with apoE genotyping, but also repeatability, reproducibility and sample storage criteria.

## 2 Material and methods

In the course of familial hypercholesterolemia screening or mixed dyslipidemia diagnosis, blood samples were collected from 40 patients in our outpatient clinic (Cardiovascular Prevention Unit, Institute of Cardiometabolism and Nutrition, La Pitié-Salpêtrière – Charles Foix University Hospital, AP-HP, Paris, France) of the Endocrinology-Metabolism Department. These patients had given their informed consent including genetic determination and *APOE* genotypes were already determined by Sanger sequencing of a PCR fragment encompassing the two polymorphic sites (rs429358 and rs7412). Venous blood was collected in gel-containing Vacutainer® tubes (Becton-Dickinson, Plymouth, UK), then allowed to clot at room temperature and centrifuged at 4500 ​rpm ​at 10 ​°C for 10 ​min, to obtain serum for routine lipid parameters analysis. Sera were immediately aliquoted and frozen at −80 ​°C until IEF assays.

The Hydragel 18 Apo E Isofocusing® (Sebia) is a qualitative RUO (Research Use Only) kit for detection and identification of the different apoE phenotypes. The method is adapted on a well-established equipment from Sebia. The references indicated on the apoE kit are temporary references since they are currently RUO products. A ready-to-use agarose gel containing ampholytes (pH gradient: 5–8) is used to perform a semi-automatic electrophoresis on Hydrasys 2 Scan® Sebia (Lisses, France), followed by a specific immunofixation with anti-apoE antiserum. Isoform bands were identified by comparison to bands of a specific control run in each gel and including E2, E3, and E4 phenotypes (Sebia, PN 4785). After reconstitution of this lyophilized control, aliquots were kept frozen at −80 ​°C. The Hydrasys 2 Scan® Sebia can perform all steps of process (migration, incubation, gel staining/destaining, drying and scanning). The “apoE isofocusing visualization” kit (Sebia, PN 4749) included an antiserum diluent, stock solutions of anti-apoE antiserum and peroxidase-labeled antibody, and reagent for revelation (TTF1 and TTF2 developing solutions).

Briefly, 20 ​μL serum were treated with 5 ​μL neuraminidase (neuraminidase type V from *Clostridium perfringens*, provided in the kit: Sigma, PN N2876) for 1 ​h at 45 ​°C. Sample was then delipidated with 25 ​μL of the delipidation solution for 1 ​h at 45 ​°C. After centrifugation for 5 ​min. ​at 5000 ​rpm, the supernatant was collected and used for IEF. Eighteen 10 ​μL-samples could be analyzed on each gel. Migration was carried out under 500 ​V until 300 Vh has been accumulated and under 1000 ​V until 100 Vh has been accumulated (final 400 Vh), at 20 ​°C (temperature controlled by Peltier effect) for about 45 ​min. A two step-immunofixation was then performed: one with the anti-human apoE polyclonal antiserum for 10 ​min. ​at 20 ​°C (temperature controlled by Peltier effect) and, after a cycle wash, another one for 10 ​min. with a peroxidase-labeled secondary antibody. The gel was then incubated with the Sebia TTF1/TTF2 substrate for 10 ​min. in order to visualize apoE isoforms. The total time to obtain the apoE status on a gel was 2h15.

The 40 sera were blindly analyzed for determination of apoE status. The repeatability of the procedure was evaluated by performing IEF of each patient sample 6-fold in the same gel. For reproducibility, each patient sample was tested 4-fold on different gels and at different days, which implied that stability of sera was tested after 3 cycles of freezing and thawing. The Sebia “control serum” (E2/E3/E4) was run in each gel, together with two sera from already typed patients.

## 3 Results

The sera from the genotyped patients included in the study allowed us to test the following isoforms: E3/E3 (18 sera), E3/E4 (16 sera), E2/E2 (4 sera) and E4/E4 (2 sera) (Table 1).

**Table 1:** Lipid parameters and apoE status of the patients.

Patients n ​= ​40		
Gender (M/F)	20/20	
Age (years)	52 ​± ​13	
Total cholesterol (mmol/L)	5.9 ​± ​1.3	
Triglycerides (mmol/L)	2.0 ​± ​1.3	
HDL-cholesterol (mmol/L)	1.37 ​± ​0.47	
LDL-cholesterol (mmol/L)	3.65 ​± ​1.24	
ApoA1 (g/L)	1.55 ​± ​0.26	
ApoB (g/L)	1.10 ​± ​0.33	
Lp(a) (g/L)	0.36 ​± ​0.38	
**Phenotype**	**Frequency (%)**	**Genotype**
E3/E3	45	ε3/ε3
E3/E4	40	ε3/ε4
E2/E2	10	ε2/ε2
E4/E4	5	ε4/ε4

**Fig. 1:**
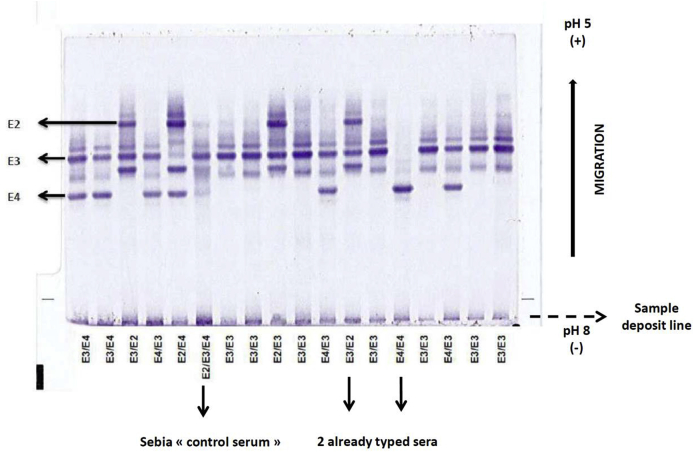
Hydragel 18 Apo E Isofocusing® (Sebia) showing different apoE isoforms from 16 patients tested. The “control serum” provided in the Sebia kit is included in each series, together with two already typed serum samples as internal controls (as an example, E3/E2 and E4/E4).

All phenotypes tested were 100% concordant to the genetic isoforms, even if all the possible phenotypes are not found in our population. An IEF gel is shown as an example (Fig. 1).

Precision of the technique was verified with the repeatability (Supplemental Fig. 1) and the reproducibility tests for the different isoforms (data not shown), since tests gave similar results. In addition, the reproducibility tests led us to perform three freezing-thawing cycles on serum samples and a 100% concordant interpretation with genotyping was maintained. Supplemental Figs. 2 and 3 show no interference of hemolysis, glucose or hypertriglyceridemia at the respective concentrations of 45.3 ​μmol/L hemoglobin, 25.1 ​mmol/L glucose and 9.5 and 4.8 ​mmol/L triglycerides for the determination of the phenotype profile. Supplemental Figs. 2 and 3 also show various apoE phenotypes.

## 4 Discussion

This study allowed us to show the advantages of this semi-automated procedure to perform apoE phenotyping, which presents an interest under some conditions where no genetic material is available. Its good precision criteria and the possibility to use frozen samples (up to 3 freezing-thawing cycles) must be put forward for the lab practice.

Our patient population differs from that of the Hirtz’s study [5]. Indeed, the latter population was constituted of patients with cognitive or behavioral disorders and presented with a high frequency (9.3%) of E4/E4 phenotypes. By contrast, in agreement with our recruitment characteristics, the patients of our study were at cardiovascular risk and 10% presented with E2/E2 phenotype, one situation that is known to be a risk factor to develop type III dyslipidemia (dysbetalipoproteinemia), with high levels of circulating intermediate density lipoproteins (IDLs) due to a decreased clearance of these lipoproteins by the liver [6]. ApoE4 also increases the risk for cardiovascular disease due to its structure which displays increased preference for large, triglyceride-rich VLDLs. In a whole, these features could explain the high frequency of E2/E2 and E3/E4 phenotypes (10 and 40%, respectively) in our population when compared with normolipidemic subjects or in general population ([9,10], Supplemental Table 1).

When compared with the initial apoE IEF procedures, the direct use of serum instead of isolated lipoproteins represents a time spare and a technical facilitation. The readability of the gel is also increased owing to the action of neuraminidase that eliminates bands corresponding to the sialylated forms of apoE, and the position of the band of each isoform is especially easy to determine by comparison with the “normal control serum” run in each gel. Another advantage is the direct immunofixation step without requiring transfer to a membrane. Finally, good repeatability and reproducibility, and the possibility to perform 3 freezing-thawing cycles on serum samples support the reliability and practicability of the test.

Thanks to our experience of the kit, we have shown a limited conservation of the neuraminidase provided in the kit (not more than 3 weeks) as well as the “normal serum” Sebia whose bands become less clear over time. For the latter reason, it is very important to include 2 pre-genotyped sera in each run to ensure proper identification of the position of the E2, E3 and E4 bands.

Other phenotyping methods are available, such as mass spectrometry [5] and ELISA. Nevertheless, in opposition with mass spectrometry that is able to detect all isoforms with a high specificity, ELISA techniques are specific for apoE4, thus not allowing a complete apoE phenotyping [7]. A recent method based on apoE-polystyrene interaction has been described, but it is again specific for apoE4 detection and does not distinguish between homozygote E4/E4 or heterozygous phenotypes containing E4 isoform [8].

Due to the specificity of the present technique (in relation with immunofixation), it is of note that other apolipoproteins present in VLDLs and IDLs cannot be seen, especially apoC (C-I, C-II, C-III), whereas they were of course present in the initial procedure using isolated lipoproteins and without immunofixation.

A limitation of our study is the low number of patients and phenotypes tested (especially, no mutated isoform was included), but the patients were selected for having had a previous genotyping and for a perfect quality and storage of their frozen sera. The impact of the sample matrix has also been tested on 4 patients, for which frozen EDTA or heparin plasma was available; only EDTA plasma gave correct apoE phenotyping, while heparin plasma has to be avoided under our experimental conditions, because of unclear interpretation (data not shown). Nevertheless, for the latter samples, storage was not well controlled before samples were frozen, so that we cannot rule out preanalytical deficiencies. Of note, Hirtz et al. [5] did not report such interference on heparin samples.

## 5 Conclusion

This qualitative, semi-automatized method could yield a novel and simple tool to phenotype apoE isoforms. Validated on a cohort of patient samples with known genotype, it could be used either for research screening and stratification of a patient cohort, or for clinical analysis when genetic material cannot be obtained. Another advantage for this test is the possibility to use a little volume of routine sera, and practice serial analysis in a short time with simplified and standardized steps. This could be a useful and supplementary tool helping the clinician in the diagnosis of dyslipidemic and neurodegenerative diseases.

## Funding sources

This research did not receive any specific grant from funding agencies in the public, commercial, or not-for-profit sectors.

## CRediT authorship contribution statement

**Randa Bittar:** Conceptualization, Investigation, Project administration, Resources, Supervision, Validation, Visualization, Writing - original draft, Writing - review & editing. **Alain Carrié:** Conceptualization, Investigation, Resources, Validation, Writing - review & editing. **Georges Nouadje:** Resources, Writing - review & editing. **Corinne Cherfils:** Investigation, Resources, Validation, Writing - review & editing. **Valérie Fesel-Fouquier:** Investigation, Validation, Writing - review & editing. **Laurence Barbot-Trystram:** Writing - review & editing. **Philippe Giral:** Writing - review & editing, Resources. **Dominique Bonnefont-Rousselot:** Conceptualization, Investigation, Project administration, Resources, Supervision, Validation, Visualization, Writing - original draft, Writing - review & editing.

## Declaration of competing interest

There are no known conflicts of interest.

## Appendix A Supplementary data

Supplemental Figure 1: Example of Hydragel 18 Apo E Isofocusing® (Sebia) showing repeatability of apoE phenotyping performed on the “control serum” provided in the Sebia kit (E2/E3/E4) and on two patient’s sera (E4/E4 and E3/E3) (n=6 for each sample).

Supplemental Figure 2: Example of Hydragel 18 Apo E Isofocusing® (Sebia) with various apoE phenotypes and study of the interference of hypertriglyceridemia for the determination of the phenotype profile.A patient EDTA plasma sample (P) with E3/E3 phenotype has been loaded with glucose (G), or with hemoglobin (H) to assess the influence of hyperglycemia and hemolysis, respectively.To test glucose interference, a stock fresh solution of 5.4 mol/L glucose has been added to the plasma (initial glucose concentration : 4.4 mmol/L) to obtain a final glucose concentration of 25.1 mmol/L.To test hemolysis interference, the plasma sample has been loaded with hemoglobin (H) by adding an hemolysate obtained by inducing osmotic lysis of the patient’s red blood cells by distilled water (v/v). The initial and final plasma hemoglobin concentrations were 8 and 45.3 μmol/L, respectively.

Supplemental Figure 3: Example of Hydragel 18 Apo E Isofocusing® (Sebia) with various apoE phenotypes and study of the interference of hypertriglyceridemia for the determination of the phenotype profile.A patient serum sample (L) with E3/E4 phenotype and hypertriglyceridemia was diluted 1/1 (v/v) with 0.15 M NaCl (L/2). Initial and final triglyceride concentrations were 9.5 and 4.8 mmol/L, respectively.
