# Supplementary figures and images for: Evaluation of a semi-automatic isoelectric focusing method for apolipoprotein E phenotyping
Source: Pract Lab Med. 2019 Dec 17;18:e00150. doi: 10.1016/j.plabm.2019.e00150 (PMC6939034; doi:10.1016/j.plabm.2019.e00150)

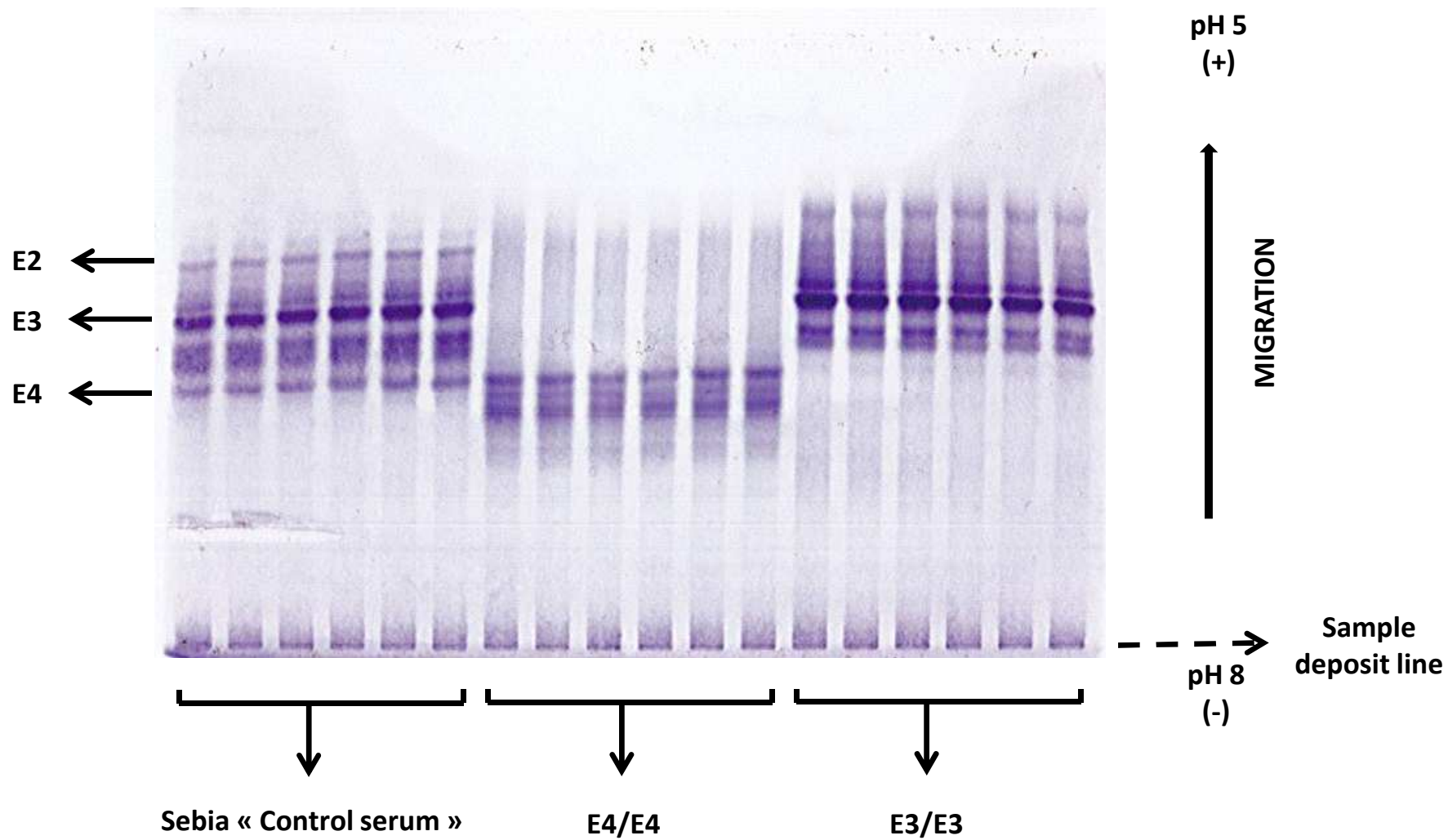

Suppl. Fig. 1

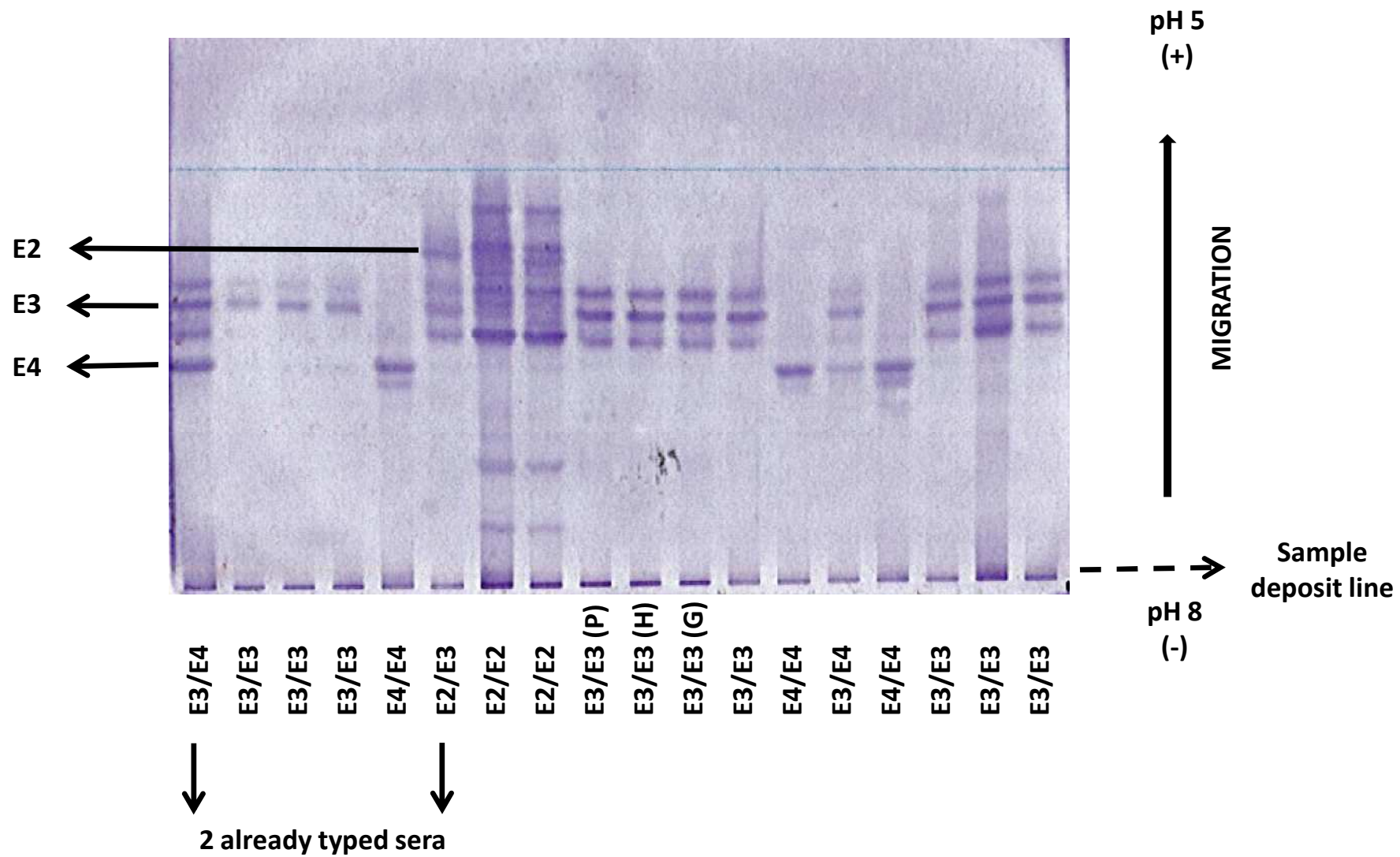

Suppl. Fig. 2

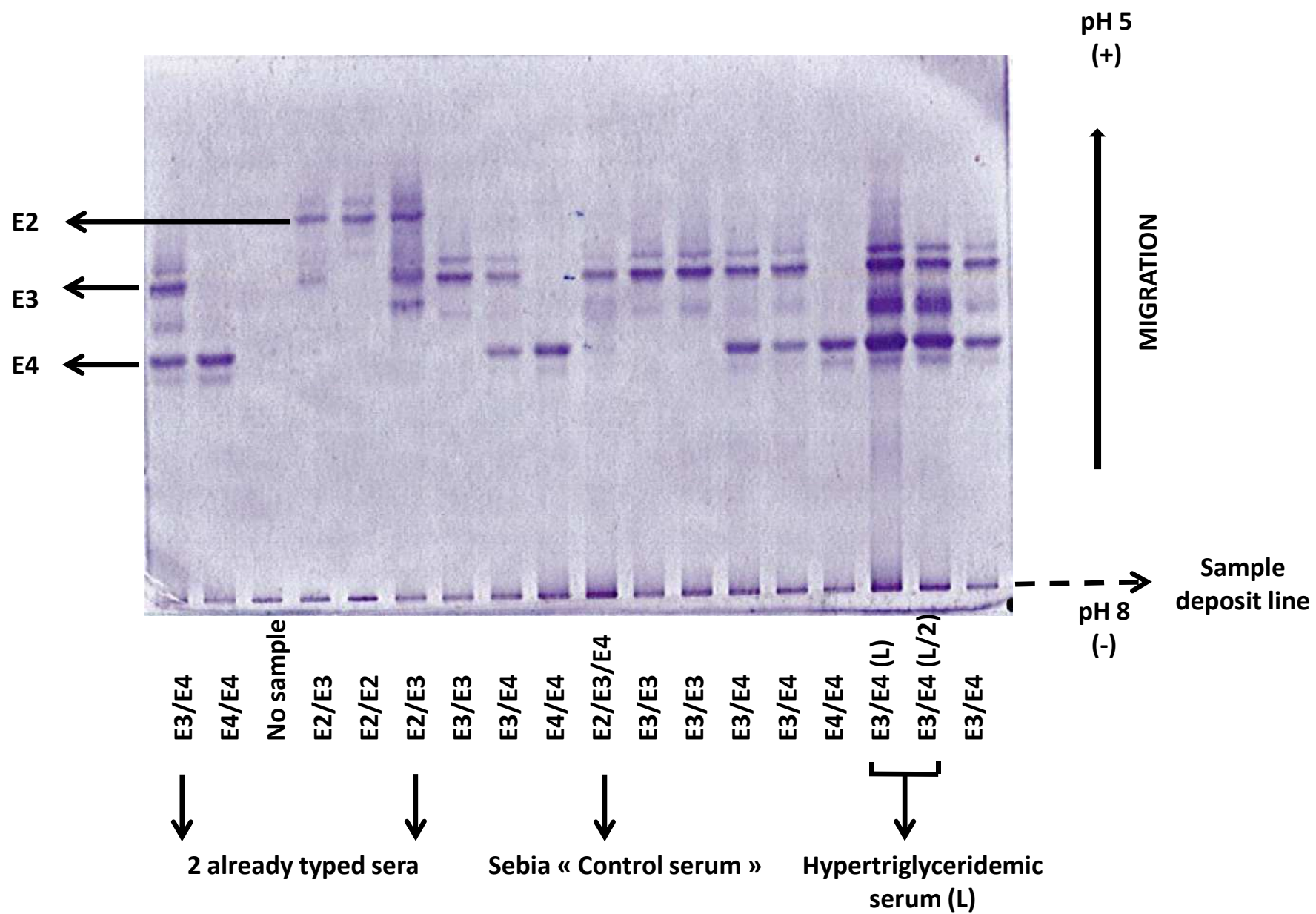

Suppl. Fig. 3

Supplement: Multimedia component 2 [file mmc2.pdf]
