# Supplementary material for: Evaluation of a semi-automatic isoelectric focusing method for apolipoprotein E phenotyping
Source: Pract Lab Med. 2019 Dec 17;18:e00150. doi: 10.1016/j.plabm.2019.e00150 (PMC6939034; doi:10.1016/j.plabm.2019.e00150)
Supplement: Multimedia component 3 [file mmc3.docx]

Supplemental Table 1. ApoE phenotype frequencies in normolipidemic patients and in general population ([9,10]).
